# A Case of Kratom Overdose in a Pediatric Patient

**DOI:** 10.1155/2020/8818095

**Published:** 2020-08-12

**Authors:** Andrew Wong, Monique Mun

**Affiliations:** Henry Ford Health System, Department of Psychiatry, 1 Ford Place, 1C-09 Detroit, Michigan, USA 48202

## Abstract

Kratom is a synthetic opioid that is federally unregulated and thus available for purchase through online retail and smoke shops in most states. Due to its availability, there is concern for misuse in the pediatric population. There is existing literature describing toxicity of kratom in adults; yet, to the best of our knowledge, there are no cases describing kratom toxicity in the pediatric population. Thus, we present the case of kratom overdose in a pediatric patient.

## 1. Introduction

Kratom is a synthetic opioid that is rapidly on the rise in the United States. Poison control centers across the United States reported the number of exposures increased tenfold from 2010 to 2015 [1]. Kratom is federally unregulated and thus available for purchase through online retail and smoke shops in most states. It is marketed as a supplement and perceived as an attractive alternative to prescribed medications. Kratom users report enhanced mood, concentration, analgesic effects, and use for preventing opioid withdrawal [2]. There is emerging literature reporting its potentially harmful effects. Due to its availability, there is concern for misuse in the pediatric population. There is existing literature describing toxicity of kratom in adults; yet, to the best of our knowledge, there are no cases describing kratom toxicity in the pediatric population. Thus, we present the case of kratom overdose in a pediatric patient.

## 2. Case

We present the case of a 15-year-old Caucasian female with no prior medical history who presented to the emergency department after ingesting 45 capsules of kratom 500 mg as a suicide attempt in context to worsening depression. The patient obtained kratom capsules from her father. After ingestion, she notified her mom who brought her to the hospital. Her parents were not aware of the potential harmful effects of kratom toxicity and thus did not store the capsules in a locked cabinet. Patient denied coingestion of other substances. On exam, patient complained of dry mouth, dizziness, restlessness, palpitations, nausea, and vomiting. Vital signs were positive for tachycardia (heart rate = 100 beats per minute). Physical exam was positive for miotic pupils and bilateral upper extremity tremors. Otherwise, the rest of the neurologic exam was unremarkable. Labs were significant for hypokalemia (*K* = 2.9) and elevated lactic acid (lactic acid = 3.2). Electrocardiogram was significant for sinus tachycardia and elevated QTc = 474. Urine drug screen was negative. Patient was placed on seizure precaution and cardiac monitoring. Patient's nausea initially did not respond to intramuscular trimethobenzamide 200 mg but resolved after one-time dose of intramuscular ondansetron 4 mg. Potassium was replaced. Approximately 14 hours after ingestion, the patient's symptoms had resolved. Behavioral health services were consulted in the emergency room and recommended the patient for inpatient psychiatric hospitalization for the suicide attempt and worsening depression. Once the patient was medical stabilized, she was transported by emergency medical services vehicle to an inpatient pediatric psychiatry unit.

## 3. Discussion

Kratom is a synthetic opioid that originates from a tropic tree (*Mitragyna speciosa*) native to Southeast Asia [2]. Its active ingredients are 7-hydroxymitragynine, a highly selective *μ* and *κ* opioid receptor agonist, and mitragynine, which acts on 5-HT2A serotonergic, alpha 2-adrenergic, and dopamine receptors. At low doses, users report stimulating effects, and at high doses, it can produce opioid-like analgesic effects [2]. Online retailers recommend a dose of 4 to 10 grams per day, with a maximum dose of 50 grams per day. Our patient ingested approximately 225 grams. Kratom toxicity has been associated with seizures, agitation, psychosis, arrhythmias, hypothyroidism, intrahepatic cholestasis, nephrotoxicity, coma, and death [2]. Cases of death related to kratom exposure almost all occurred in patients 20 years and older, with the exception of one case reporting death in a 17-year-old who coingested over-the-counter cold medications and benzodiazepines [1, 3]. No causality of death has been established with kratom, though coingestion with other drugs of abuse was found in most cases of deaths related to kratom exposure [1].

In the adolescent population, kratom is an emerging drug of abuse. A study from poison control centers in the United States from 2011 to 2017 reported that among adolescents age 13-19 years old, most exposure was due to intentional abuse/misuse (75.9%) and suspected suicide (10.2%) [1]. In addition, site of ingestion of kratom occurred mostly at home (75.9%) [1]. Kratom is attractive in that it produces mood-altering and opioid-like effects yet can be viewed “safer” compared to prescribed medications or other drugs of abuse due to its label as a supplement. Furthermore, parents who use kratom may not be as vigilant in storing it away in a locked medication cabinet due to its perceived safety or lack of knowledge about its potentially harmful effects.

Infants born to pregnant mothers who used kratom during pregnancy were found to exhibit neonatal abstinence syndrome. There have been at least six cases of kratom-associated neonatal abstinence syndrome (KANAS), occurring 6-96 hours after birth. KANAS presents as difficulty feeding, excessive sucking, emesis, jitteriness, irritability, hypertonia, tremors, sneezing, facial excoriations, and high-pitched cry [4]. In addition, electrolyte abnormalities, elevated aspartate aminotransferase (AST) or alanine transaminase (ALT) levels (>100), hypoglycemia, and dyspnea can occur in KANAS [1].

## 4. Conclusion

In summary, kratom is a substance that is widely accessible yet is potentially harmful when misused or ingested in large amounts. Because it is unregulated and sold as a supplement, it can be misused in the pediatric population due its accessibility, lack of knowledge of its potentially harmful effects from parents, and perceived safety as a supplement.
